# The Effects of Calcination Process Parameters on RHA Reactivity and Mortar Mechanical Properties

**DOI:** 10.3390/ma18133129

**Published:** 2025-07-02

**Authors:** Jianrui Ji, Lihui Li, Lei Quan, Bo Tian, Panpan Zhang, Sili Li

**Affiliations:** 1 School of Civil Engineering, Chongqing Jiaotong University, Chongqing 400074, China; jijianrui@mails.cqjtu.edu.cn (J.J.); b.tian@rioh.cn (B.T.); 2 Research Institute of Highway, Ministry of Transport, Beijing 100088, China; lilihui0451@163.com (L.L.); l.quan@rioh.cn (L.Q.); 18293198316@163.com (S.L.)

**Keywords:** rice husk ash, calcination process, activity, mortar, mechanical properties

## Abstract

The insufficient optimization of calcination process parameters severely restricts the enhancement of rice husk ash (RHA) volcanic ash activity. In this study, an intelligent muffle furnace was used for multi-parameter coupled regulation, combined with microscopic characterization techniques, to elucidate the effects of temperature, cooling mode, heating rate, and holding time on the reactivity of RHA. The results showed that the effect of calcination temperature on the volcanic ash activity of RHA was dominant. RHA calcined at 600–700 °C showed a honeycomb porous structure, displayed broad amorphous SiO_2_ diffraction peaks and up to 95% content of SiO_2_, and exhibited the best volcanic ash activity. The increased crystallinity of RHA calcined at 800 °C led to a decrease in its activity. The subcooling treatment with distilled water effectively rebuilt the lamellar structure, reduced the crystallinity, and enhanced the reactivity. The samples incorporated with 600 °C calcined RHA showed higher compressive strength at 3 days compared to 800 °C calcined RHA.

## 1. Introduction

Building a low-carbon society has become a strategic priority in China and the world. As the most used and widely used construction material in the world, the mass production of cement and concrete consumes a large amount of natural resources and increases the emission of carbon dioxide. The production of one ton of cement releases about one ton of carbon dioxide, which accounts for 8% of the total global carbon dioxide emissions and poses a great danger to the environment [1,2,3]. Therefore, it is necessary to use supplementary cementitious materials (SCMs) to replace part of the cement to reduce the energy consumption and CO_2_ emissions associated with cement production [4,5].

Rice husk is an unavoidable by-product of the rice industry, and China, as the world’s number one country in terms of demand for agricultural products, leaves about 40 million tons of rice husk as waste from its annual production [6]. Therefore, there is an urgent need for its effective utilization in order to avoid environmental pollution. In contrast, rice husk ash (RHA), which is burned under certain combustion conditions, contains a large amount of highly reactive amorphous SiO_2_ and is very suitable as an external admixture for cement-based building materials [7,8]. This way of treating rice husk significantly increases its added value, which not only protects the environment and saves energy, but also improves the performance of cement-based materials.

The effect of RHA on the properties of cementitious materials has been extensively studied. Because RHA contains a large amount of amorphous SiO_2_ [9], it gives rise to a certain volcanic ash activity. The volcanic ash reaction of RHA reduces the CH content [10], which leads to the formation of more C-S-H gels and thus improves the properties of cementitious materials. However, the volcanic ash activity of RHA is strongly influenced by the calcination process. It has been shown that combustion at 800–1000 °C leads to the presence of crystalline SiO_2_ of square and scale quartz in RHA, which reduces its reactivity [11]. Sung-Hoon Kang et al. [12] demonstrated that RHA burned at 500–700 °C had the highest silica content. Nair et al. [13] found that longer combustion times at 500–700 °C increased the reactivity of RHA, while shorter times resulted in higher carbon content. Shatat [14] achieved RHA with 86% amorphous SiO_2_ by burning at 600 °C for 2 h, while Zhu [15] obtained RHA with 93.10% amorphous SiO_2_ by burning at 800 °C for 0.5 h. Mohseni [16] burned RHA at a controlled rate to 700 °C for 3.5 h, resulting in RHA with 91.15% amorphous SiO_2_. However, Wang [17] found that burning RHA at 600 °C only obtained 84% SiO_2_, with a lower amorphous SiO_2_ content of 55.87% and a crystalline SiO_2_ content of 28.13%. Additionally, Rego et al. [18] observed a positive correlation between RHA reactivity and SiO_2_ content. These studies highlight the importance of controlling the combustion parameters to achieve desired amorphous SiO_2_ content in RHA. Sung-Hoon Kang et al. [12] showed that the combustion of RHA at less than or equal to 700 °C produces a large amount of amorphous SiO_2_, and its combustion at 500–700 °C makes the highest silica content. Nair et al. [13] pointed out that burning at 500–700 °C for more than 12 h made their RHA highly reactive, while short-time combustion (15–360 min) resulted in RHA containing a high carbon content. M.R. Shatat [14] prepared RHA containing 86% amorphous SiO_2_ by burning rice husk at 600 °C for 2 h. Huajun Zhu et al. [15] prepared RHA with 93.10% amorphous SiO_2_ by burning rice husk in a muffle furnace at 800 °C for 0.5 h. A study by Ehsan Mohseni et al. [16] showed that burning RHA at a constant rate of 200 °C per hour for 3.5 h to 700 °C and cooled for 1 h resulted in RHA containing 91.15% amorphous SiO_2_. In contrast, Wang et al. [17] found that burning RHA at 600 °C obtained 84% SiO_2_, with an amorphous SiO_2_ content of 55.87% and a crystalline SiO_2_ content of 28.13%. In addition, Rego [18] et al. compared the volcanic ash activity of two RHAs with high and low amorphous SiO_2_ content and showed that the RHA reactivity and SiO_2_ content were positively correlated. Therefore, the content of amorphous SiO_2_ has an important effect on the volcanic ash activity of RHA in cementitious materials, which in turn is closely related to its calcination process.

In summary, the volcanic ash activity of rice husk ash (RHA) is highly dependent on the regulation of SiO_2_ crystallization behavior during the calcination process, with inadequate optimization of calcination parameters significantly restricting its practical application in engineering. While previous studies have confirmed the correlation between RHA reactivity and calcination conditions, considerable discrepancies remain regarding the critical temperature thresholds (e.g., the amorphous SiO_2_ content at 500 °C versus 800 °C). Furthermore, limited research has been conducted on the effects of heating rate and cooling method on RHA reactivity. Existing studies predominantly focus on either material characteristics (e.g., RHA crystallinity) or macroscopic properties (e.g., mortar strength) in isolation, lacking an integrated analysis that bridges RHA reactivity with the mechanical performance of cementitious composites.

To address these challenges, this study employs a multi-parameter coupled experimental design to systematically investigate the effects of calcination parameters—including temperature, heating rate, holding time, and cooling method—on RHA reactivity and its influence on the mechanical properties of mortar. The findings provide a theoretical foundation for optimizing RHA calcination processes to enhance the volcanic ash activity, contributing to the sustainable utilization of RHA in cement-based materials.

## 2. Material and Methods

### 2.1. Material

The cement in this study was P·O 42.5 ordinary silicate cement produced by China General Research Institute of Building Materials Science (Beijing, China), and its chemical composition is shown in Table 1. Rice husk ash was prepared using rice husks provided by the rice processing plant in the suburbs of Heilongjiang Province as raw materials: After cleaning with clean water to remove surface dirt, it was dried in an oven at 105 °C for 24 h to remove moisture, and then the raw materials were processed in a muffle furnace (type KSZN-K8) to prepare rice husk ash. The fine aggregate was natural river sand with a fineness modulus of 2.96; the mixing water was tap water.

### 2.2. Method

The KSZN-K8 intelligent muffle furnace produced by Zhengzhou Sanbo Coal Measurement and Control Instrumentation Co., Ltd. (Zhengzhou, China) was used to study the variation rule of the composition and morphology of rice husk ash by changing the burning temperature, heating rate and residence time.

It was shown that the best volcanic ash activity could be obtained by calcining rice husk below 780 °C [19]. The scorching temperatures were set to four specific temperatures, 600 °C, 700 °C, 800 °C, and 900 °C, and the constant temperature times were set to 1 h and 2 h [20]; the specific scorching methods are shown in Table 2. The microscopic morphological characteristics of rice husk ash under different burning methods were measured by field emission electron scanning microscopy (SEM), and X-ray diffractometry (XRD) was used to determine the structure and physical phase of rice husk ash, and the mass fraction of elements contained in rice husk ash was analyzed semi-quantitatively by energy-dispersive X-ray spectrometry (EDS). In this study, the ratio of water-to-cement (w/c) was 0.4 for all rice husk ash cement mortar specimens. Specimens were mixed with different conditions of calcined rice husk ash, according to the standard method [21] for testing the basic properties of construction mortar for its mechanical properties, as shown in Table 3. All hardened samples (40 × 40 × 160 mm) were demolded after 1 d of curing, and further curing was performed in a water-conditioned room at a temperature of 23 ± 2 °C and relative humidity of 60 ± 5% until specified curing ages were reached.

## 3. Results and Discussion

### 3.1. RHA Micro-Morphological Analysis

**Figure 1:**
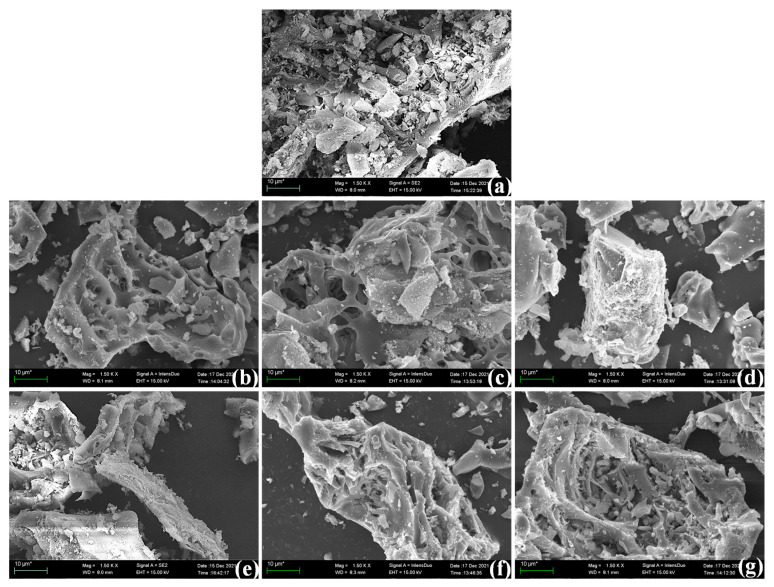
Variation in microscopic morphology of rice husk ash (from plate-like to honeycomb to spheroidal) under different calcination processes: (**a**) calcination mode 1; (**b**) calcination mode 2; (**c**) calcination mode 3; (**d**) calcination mode 4; (**e**) calcination mode 5; (**f**) calcination mode 6; (**g**) calcination mode 7.

This study conducted scanning electron microscopy (SEM) analysis to investigate the microstructural characteristics of rice husk ash (RHA) obtained under different calcination conditions. Figure 1 presents the morphological features of RHA particles subjected to various calcination processes. The results indicate that RHA particles predominantly exhibit polygonal and angular morphologies, along with distinctly porous surfaces and a honeycomb-like structure. This unique microstructure is attributed to the combustion-induced decomposition and volatilization of organic matter, which facilitates the formation of a highly porous, interconnected network. Consequently, the resulting RHA demonstrates a high specific surface area and porosity, key properties that contribute to its enhanced volcanic ash activity [22].

As shown in Figure 1a–d, the degree of particle fragmentation in rice husk ash (RHA) progressively increases with rising calcination temperature. Specifically, at 600 °C, RHA particles exhibit minimal fragmentation and maintain a relatively intact structure. However, at 700 °C and 800 °C, the degree of breakage increases, and by 900 °C, RHA particles experience the most severe structural degradation. These findings indicate that higher calcination temperatures induce significant microstructural changes in RHA.

At 600 °C, the RHA surface features interwoven nanoscale platelets, which are loosely aggregated clusters of amorphous SiO_2_. This highly reactive structure aligns with previous studies, confirming that low-temperature calcination facilitates the formation of amorphous SiO_2_, thereby enhancing volcanic ash activity [23,24]. As the temperature increases to 800 °C, these fine flakes gradually collapse, the surface roughness intensifies, and an interconnected honeycomb structure appears, increasing the contact area with the outside world. This transformation is likely due to the progressive volatilization of organic matter during calcination, which alters the surface morphology of RHA. The formation of this honeycomb structure is consistent with findings reported in the literature, highlighting the critical role of calcination temperature and organic matter decomposition in shaping RHA’s surface topology [24].

At 900 °C, the interconnected structure nearly disappears, and the particles transition into irregular, quasi-spherical morphologies, indicating that amorphous SiO_2_ has undergone crystallization into a more thermodynamically stable phase. This phenomenon has been widely documented in prior research [25], demonstrating that high-temperature calcination promotes SiO_2_ crystallization, leading to a substantial decline in pozzolanic reactivity. Consequently, RHA calcined at excessively high temperatures exhibits significantly reduced reactivity as a supplementary cementitious material, thereby diminishing its effectiveness as a pozzolanic additive in concrete.

As observed in Figure 1b,c,f,g, the heating rate and holding time exert minimal influence on the surface morphology of RHA when calcined at 800 °C, where the material consistently exhibits a characteristic interconnected structure. This suggests that at 800 °C, variations in heating rate and holding time do not significantly alter the microstructural features of RHA. However, given the narrow range of heating rates and holding times employed in this study, the findings may not encompass all possible conditions. A more comprehensive investigation covering a broader spectrum of parameters would provide deeper insights into the effects of heating rate and holding time on the microstructure and performance of RHA.

In contrast, Figure 1e reveals that RHA subjected to subcooled distilled water treatment exhibits a well-defined layered structure, indicating that the cooling method plays a significant role in determining the final morphology of RHA. This phenomenon can be attributed to the kinetic suppression of crystal growth during rapid cooling, wherein water quenching induces a sudden increase in the viscosity of molten SiO_2_, thereby inhibiting the directional arrangement of crystallites. As a result, the supercooling treatment modifies the aggregation behavior of SiO_2_, promoting the formation of a more ordered structure.

**Figure 2:**
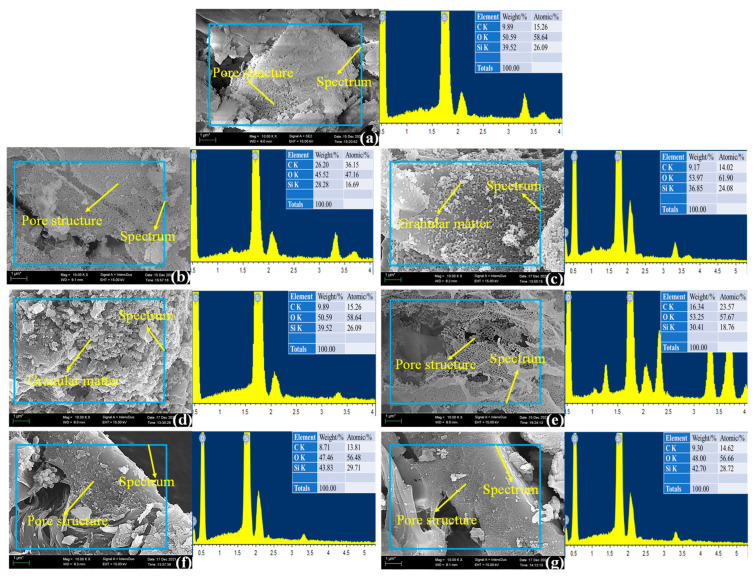
Energy spectra of rice husk ash under different calcination processes: (**a**) first calcination method; (**b**) second calcination method; (**c**) third calcination method; (**d**) fourth calcination method; (**e**) fifth calcination method; (**f**) sixth calcination method; (**g**) seventh calcination method.

Figure 2 presents high-magnification (10,000×) SEM images of RHA along with elemental energy-dispersive X-ray spectrometry (EDS) analysis. The results indicate that RHA predominantly consists of oxygen (O) and silicon (Si) elements, confirming that silicon dioxide (SiO_2_) is the primary component. Additionally, a small amount of carbon (C) is detected, which can be attributed to incomplete combustion during the calcination process.

At higher magnifications, numerous micropores are observed on the RHA surface. However, when the calcination temperature reaches 800 °C, the RHA surface exhibits a significant presence of granular structures (Figure 2c). This phenomenon becomes more pronounced at 900 °C, where a substantial number of granular deposits are evident on the surface (Figure 2d). These observations align with the microstructural changes identified in Figure 1 at lower magnifications and suggest that the amorphous SiO_2_ in RHA undergoes crystallization at elevated temperatures, forming crystalline silica.

Notably, when RHA calcined at 800 °C undergoes distilled water quenching, these granular structures disappear, and the surface retains a highly porous morphology (Figure 2e). This further substantiates that rapid cooling effectively suppresses crystal growth and significantly modifies the microstructure of RHA.

### 3.2. RHA Composition Analysis

**Figure 3:**
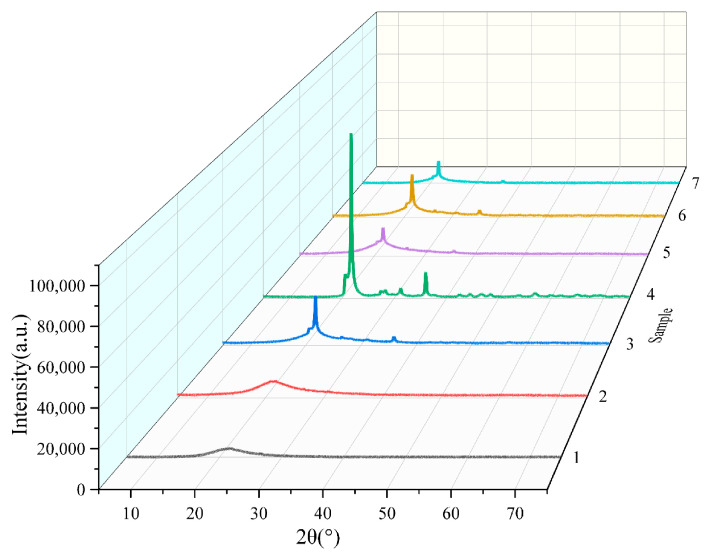
XRD patterns of rice husk ash under different calcination processes.

The X-ray diffraction (XRD) patterns of RHA obtained under different calcination conditions are presented in Figure 3. As observed in curves 1 and 2, no distinct crystalline diffraction peaks appear in the XRD spectra. Instead, a broad hump-shaped scattering curve is centered at 2θ ≈ 22.5°, extending within the range of 2θ = 15° to 30°. This characteristic peak is a well-established signature of amorphous SiO_2_ [26].

Most importantly, the proportion of amorphous SiO_2_ in RHA is a key determinant of its pozzolanic reactivity, which directly influences its effectiveness as a supplementary cementitious material (SCM) in concrete [27]. These findings confirm that RHA obtained from calcination at 600–700 °C predominantly consists of amorphous SiO_2_, ensuring its potential for high reactivity in cementitious applications.

As the calcination temperature increased to 800 °C, a narrow and sharp peak at 2θ ≈ 22.5° started to appear in curves 3, 5, 6, and 7. This indicates that the SiO_2_ underwent a crystalline transformation, and some of the amorphous SiO_2_ converted into crystalline SiO_2_. Furthermore, when the rice husk ash was subcooled using distilled water, the peak at 2θ ≈ 22.5° was noticeably reduced in curve 5. This suggests that the distilled water subcooling treatment significantly increased the content of amorphous SiO_2_ in the ash. Comparison of curves 3, 6, and 7 reveal that the rate of temperature and constant temperature time has less effect on the physical form of silica in rice husk ash. When the temperature is increased to 900 °C, curve 4 shows a higher intensity spike at 2θ≈22.5°, which indicates that the crystallinity of silica continues to increase, mainly in the form of crystalline silica. This also explains the change in the rice husk ash from the original slab-like structure to irregular sphere-like structure shown in Figure 1 and Figure 2. In addition, several sets of spikes with relatively low intensity appear in the region from 2θ = 28° to 48°, which may be the result of the crystalline transformation of other impurities mixed in the rice husk at high temperatures. In conclusion, calcination of rice husk at temperatures above 700 °C results in a crystalline transformation of SiO_2_ and an increase in crystallinity.

**Figure 4:**
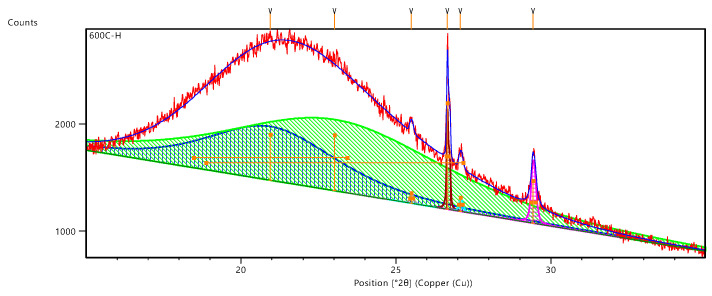
Crystallinity fit of the sample under calcination at 600 °C.

To further quantify the content of amorphous SiO_2_ in rice husk ash, the XRD patterns of rice husk ash obtained under different calcination processes were fitted to calculate its crystallinity, and the fitting schematic is shown in Figure 4, where the crystallinity of rice husk ash was calculated according to Equation (1):

(1) $$X_{c}=\frac{\sum I_{c}}{\sum I_{c}+I_{a}}\times 100\%$$

where $X_{c}$ is the crystallinity of rice husk ash, $I_{c}$ is the peak area of crystalline silica, and $I_{a}$ is the peak area of amorphous SiO_2_.

**Figure 5:**
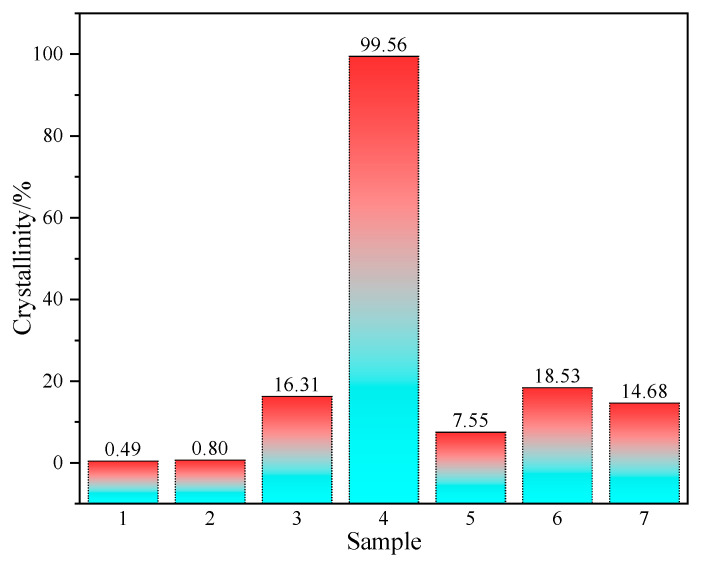
Crystallinity of rice husk ash under different calcination processes.

Figure 5 illustrates the crystallinity of rice husk ash obtained through various calcination processes. The results indicate that the crystallinity of silicon dioxide within the ash is nearly zero following calcination at temperatures ranging from 600 °C to 700 °C. This finding suggests that, within this temperature range, silicon dioxide predominantly exists in an amorphous state in rice husk ash. Such results are consistent with previous studies, which demonstrate that lower calcination temperatures facilitate the preservation of the amorphous structure of rice husk ash, thereby enhancing its volcanic ash activity [13,28]. In particular, within the temperature interval of 500 °C to 700 °C, a higher proportion of silicon dioxide remains in an amorphous form, consequently augmenting its reactivity with cementitious materials [13].

When the calcination temperature increases to 800 °C, the crystallinity of Sample 3 reaches 16.31%, indicating that a phase transition from amorphous to crystalline SiO_2_ occurs at this temperature. However, the crystalline SiO_2_ content remains below 20%, suggesting that partial crystallization has taken place, but a significant portion of amorphous SiO_2_ is still retained. This observation aligns with previous studies, which have reported that amorphous SiO_2_ in RHA begins to transform into crystalline phases at 800 °C, leading to a substantial reduction in volcanic ash activity [24].

Interestingly, when rice husk ash was subjected to distilled water subcooling treatment, its crystallinity significantly decreases to 7.55%, indicating that rapid quenching effectively inhibits high-temperature (800 °C) crystallization, thereby preserving a greater proportion of amorphous SiO_2_. This finding provides a new perspective on enhancing the pozzolanic reactivity of RHA. A similar phenomenon was reported by Nair et al., who compared rapid cooling (removal of samples from the furnace immediately after combustion) with slow cooling (allowing samples to cool naturally within the furnace). Their results demonstrated that rapid cooling increases the proportion of amorphous SiO_2_ in RHA, thereby improving its volcanic ash activity [13].

However, the study also reveals that the heating rate and holding time have a relatively minor effect on the crystallinity of RHA, with Sample 6 and Sample 7 exhibiting crystallinities of 18.53% and 14.68%, respectively. This finding differs from some reports in the literature. For instance, S. Chandrasekhar et al. [29] suggested that heating rate and holding time significantly influence the physical structure of RHA, particularly the retention of amorphous SiO_2_, with longer holding times and slower heating rates reducing amorphous SiO_2_ content. However, in this study, the impact of these parameters on crystallinity appears limited, possibly due to specific experimental conditions and the intrinsic properties of the rice husk ash used, which warrants further investigation.

When the calcination temperature increases to 900 °C, the crystallinity of Sample 4 rises sharply to 99.56%, indicating that nearly all SiO_2_ has transitioned into its crystalline form. This result confirms that high-temperature calcination causes a near-complete loss of volcanic ash activity in RHA. This finding is consistent with the study by Wang et al. [17], who reported that RHA calcined at temperatures exceeding 900 °C exhibits negligible reactivity and provides little benefit in enhancing the performance of cementitious materials. Consequently, RHA produced at temperatures above 900 °C should be avoided as a supplementary cementitious material due to its severely diminished volcanic ash activity.

To further determine the content of SiO_2_ in the ash obtained after calcination at 600 °C to 800 °C, XRF tests were performed on Samples 1, 2, and 3, and the experimental results are shown in Table 4. It can be seen that the main component of the rice husk ash is SiO_2_, which accounts for about 95% of the total mass of rice husk, and its content is not affected by the calcination temperature, and the test results are consistent with the conclusion of Feng [30]. The content of metal oxides and phosphides is low, indicating that the rice husk used in this study had a low content of impurities.

### 3.3. Effect of Crystallinity of RHA on Mechanical Properties of Mortar

**Figure 6:**
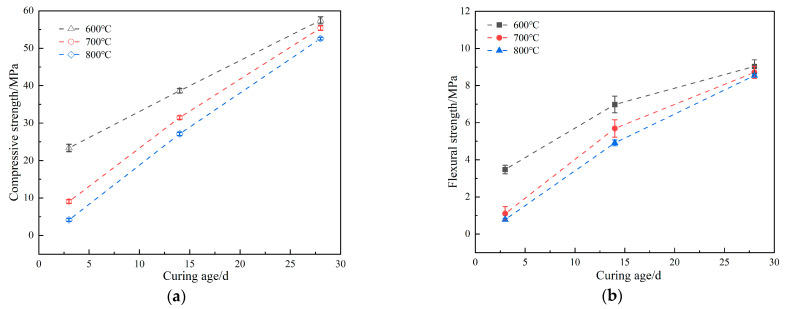
Effect of rice husk ash at different calcination temperatures on the mechanical properties of mortar. (**a**) Compressive strength. (**b**) Flexural strength.

The analysis from the aforementioned research indicates that temperature has the most significant impact on the physical morphology of silicon dioxide in rice husk ash, with the ash obtained from calcination at temperatures between 600 °C and 800 °C exhibiting higher reactivity. Consequently, this study incorporated rice husk ash calcined at 600 °C, 700 °C, and 800 °C into cement mortar to assess its effects on the compressive strength and flexural strength of the mortar. The results are presented in Figure 6. As shown in Figure 6a,b, at curing ages of 3, 14, and 28 days, both compressive and flexural strength decrease with increasing RHA calcination temperature, which corresponds to an increase in its crystallinity. At an early curing age (3 days), mortar containing 800 °C-calcined RHA (crystallinity: 16.31%) exhibited 25.9% lower compressive strength and 29.8% lower flexural strength compared to mortar with 600 °C-calcined RHA (crystallinity: 0.49%). By 28 days, these strength reductions were less pronounced, with compressive and flexural strength decreasing by only 4.3% and 3.3%, respectively.

These results demonstrate that RHA calcined at 600 °C exhibits the greatest enhancement in mortar strength, particularly at early ages (3 days). This is primarily attributed to the fact that RHA obtained at 600 °C remains predominantly amorphous, retaining high pozzolanic reactivity. In cementitious systems, amorphous SiO_2_ in RHA undergoes a secondary hydration reaction with calcium hydroxide (Ca(OH)_2_) released during cement hydration, forming additional low-calcium calcium silicate hydrate (C-S-H) gel, which significantly contributes to the early-age strength development of the mortar.

However, as calcination temperature increases, the amorphous SiO_2_ in RHA gradually transitions into a crystalline phase, leading to a loss of pozzolanic reactivity and a corresponding decline in mortar strength. This trend is consistent with the findings of Xu et al. [24], who reported that mortar incorporating 600 °C-calcined RHA exhibited significantly higher compressive strength than the control group, while 700 °C- and 800 °C-calcined RHA resulted in reduced compressive strength. Furthermore, XRD analysis confirmed that RHA calcined at 600 °C had lower crystallinity, further supporting the correlation between calcination temperature, crystallinity, and reactivity.

This study establishes a foundational framework for correlating calcination temperature, crystallinity, and mechanical properties, providing theoretical support for the precise application of RHA in low-carbon cementitious materials.

## 4. Conclusions

(1) When the calcination temperature was at 600–700 °C, the fragmented flakes on the surface of rice husk ash gradually became concave, and their roughness gradually increased into a honeycomb structure with cross-linking, and the XRD results showed that the silica in the rice husk ash was amorphous, and the silica content was as high as 95%, which provided a high activity.
(2) When the calcination temperature was 800 °C, more granular material appeared on the surface of rice husk ash, and the crystallinity was 16.3%. But at the calcination temperature of 900 °C, the cross-linked honeycomb structure of the rice husk ash disappeared and became irregular sphere-like, with a crystallinity of 99.6%, indicating that the crystalline transformation of SiO_2_ occurred when the calcination temperature reached 800 °C, and all of the amorphous SiO_2_ was transformed into crystalline SiO_2_ when it reached 900 °C, and its activity was lost.
(3) The subcooled treatment with distilled water altered the microstructure of the rice husk ash, resulting in a crystallinity decrease from 16.3% under natural cooling to 7.5%. However, modifications in the heating rate or isothermal duration had little effect on the micro-morphology and crystallinity of the rice husk ash, indicating that the influence of calcination process parameters on the activity of rice husk ash followed this order: calcination temperature, cooling treatment, heating rate, and constant temperature time.
(4) The compressive and flexural strengths of the rice husk ash cement mortar decreased with the increase in the calcined rice husk temperature, corresponding to a rise in crystallinity. When compared with the rice husk ash calcined at 800 °C, the rice husk ash calcined at 600 °C significantly improved the early mechanical properties of the cement mortar.

## Figures and Tables

**Table 1:** Chemical composition of P·O 42.5 cement.

Composition wt%	CaO	SiO_2_	Al_2_O_3_	MgO	Fe_2_O_3_	P_2_O_5_	TiO_2_	K_2_O	Na_2_O	SO_3_	Loss
Cement	62.4	21.35	4.76	3.06	3.13	0.49	—	0.21	0.54	2.52	1.54

**Table 2:** Preparation process of rice husk ash.

No.	Starting Temperature/°C	Maximum Temperature/°C	Constant Temperature Time/h	Temperature Rise Rate (°C/h)	Cooling Method
1	room temperature	600	2	300	natural cooling
2	room temperature	700	2	300	natural cooling
3	room temperature	800	2	300	natural cooling
4	room temperature	900	2	300	natural cooling
5	room temperature	800	2	300	distilled water subcooling
6	room temperature	800	2	400	natural cooling
7	room temperature	800	1	300	natural cooling

**Table 3:** Mix proportions.

Mix	w/c	Cement/g	Sand/g	Water/g	Rice Husk Ash/g
600 °C	0.4	900	2000	400	100
700 °C	0.4	900	2000	400	100
800 °C	0.4	900	2000	400	100

**Table 4:** Chemical composition of rice husk ash/%.

Temperature of Combustion	SiO_2_	Al_2_O_3_	Na_2_O	P_2_O_5_	CaO	MgO
600 °C	94.57	1.21	0.21	0.22	0.35	0.92
700 °C	94.74	1.41	0.22	0.21	0.33	0.85
800 °C	94.78	1.24	0.21	0.22	0.36	0.80

## Data Availability

The original contributions presented in this study are included in the article. Further inquiries can be directed to the corresponding author.

## References

[1] Antiohos S.K., Tapali J.G., Zervaki M., Sousa-Coutinho J., Tsimas S., Papadakis V.G. (2013). Low embodied energy cement containing untreated RHA: A strength development and durability study. Constr. Build. Mater..

[2] Aprianti E., Shafigh P., Bahri S., Farahani J.N. (2015). Supplementary cementitious materials origin from agricultural wastes–A review. Constr. Build. Mater..

[3] Prasittisopin L., Trejo D. (2015). Hydration and phase formation of blended cementitious systems incorporating chemically transformed rice husk ash. Cem. Concr. Compos..

[4] Zunino F., Lopez M. (2016). Decoupling the physical and chemical effects of supplementary cementitious materials on strength and permeability: A multi-level approach. Cem. Concr. Compos..

[5] Alex J., Dhanalakshmi J., Ambedkar B. (2016). Experimental investigation on rice husk ash as cement replacement on concrete production. Constr. Build. Mater..

[6] Shen J., Liu X., Zhu S., Zhang H., Tan J. (2011). Effects of calcination parameters on the silica phase of original and leached rice husk ash. Mater. Lett..

[7] Huang H., Gao X., Wang H., Ye H. (2017). Influence of rice husk ash on strength and permeability of ultra-high performance concrete. Constr. Build. Mater..

[8] Agwa I.S., Omar O.M., Tayeh B.A., Abdelsalam B.A. (2020). Effects of using rice straw and cotton stalk ashes on the properties of lightweight self-compacting concrete. Constr. Build. Mater..

[9] Rashid M., Molla M.K., Ahmed T.U. (2010). Durability of mortar in presence of Rice Husk Ash. World Acad. Sci. Eng. Technol..

[10] Monteiro P.J.M., Mehta P.K. (2006). Concrete: Microstructure, Properties and Materials.

[11] Cizer Ö., Van Balen K., Elsen J., Van Gemert D. (2006). Carbonation and Hydration of Calcium Hydroxide and Calcium Silicate Binders with Rice Husk Ash. Proceedings of the 2nd International RILEM Symposium.

[12] Kang S.-H., Hong S.-G., Moon J. (2019). The use of rice husk ash as reactive filler in ultra-high performance concrete. Cem. Concr. Res..

[13] Nair D.G., Fraaij A., Klaassen A.A.K., Kentgens A.P.M. (2008). A structural investigation relating to the pozzolanic activity of rice husk ashes. Cem. Concr. Res..

[14] Shatat M.R. (2016). Hydration behavior and mechanical properties of blended cement containing various amounts of rice husk ash in presence of metakaolin. Arab. J. Chem..

[15] Zhu H., Liang G., Xu J., Wu Q., Zhai M. (2019). Influence of rice husk ash on the waterproof properties of ultrafine fly ash based geopolymer. Constr. Build. Mater..

[16] Mohseni E., Kazemi M.J., Koushkbaghi M., Zehtab B., Behforouz B. (2019). Evaluation of mechanical and durability properties of fiber-reinforced lightweight geopolymer composites based on rice husk ash and nano-alumina. Constr. Build. Mater..

[17] Wang W., Meng Y., Wang D. (2017). Effect of Rice Husk Ash on High-Temperature Mechanical Properties and Microstructure of Concrete. Kem. U Ind..

[18] Rego J.H.S., Nepomuceno A.A., Figueiredo E.P., Hasparyk N.P., Borges L.D. (2015). Effect of Particle Size of Residual Rice-Husk Ash in Consumption of Ca(OH)2. J. Mater. Civ. Eng..

[19] Suomie R.W., Mishra B.P., Das S. (2025). Performance of rice husk ash (RHA) and recycled coarse aggregate (RCA) for sustainable concrete: A review. Next Mater..

[20] Su Q., Xu J. (2024). Influence of waste glass and rice husk ash on the dynamic compressive properties and variable-angle shear characteristics of concrete after high-temperature treatment: Experimental and numerical study. Structures.

[21] (2009). Standard for Test Methods of Basic Properties of Construction Mortar.

[22] Bui D.D. (2001). Rice Husk Ash a Mineral Admixture for High Performance Concrete.

[23] Vieira A.P., Toledo Filho R.D., Tavares L.M., Cordeiro G.C. (2020). Effect of particle size, porous structure and content of rice husk ash on the hydration process and compressive strength evolution of concrete. Constr. Build. Mater..

[24] Xu W., Lo T.Y., Memon S.A. (2012). Microstructure and reactivity of rich husk ash. Constr. Build. Mater..

[25] Ye G., Huang H., Van Tuan N. (2018). Rice Husk Ash. Properties of Fresh and Hardened Concrete Containing Supplementary Cementitious Materials: State-of-the-Art Report of the RILEM Technical Committee 238-SCM, Working Group 4.

[26] Rodríguez de Sensale G., Rodríguez Viacava I. (2018). A study on blended Portland cements containing residual rice husk ash and limestone filler. Constr. Build. Mater..

[27] Wang J., Xiao J., Zhang Z., Han K., Hu X., Jiang F. (2021). Action mechanism of rice husk ash and the effect on main performances of cement-based materials: A review. Constr. Build. Mater..

[28] Zareei S.A., Ameri F., Dorostkar F., Ahmadi M. (2017). Rice husk ash as a partial replacement of cement in high strength concrete containing micro silica: Evaluating durability and mechanical properties. Case Stud. Constr. Mater..

[29] Chandrasekhar S., Pramada P.N., Majeed J. (2006). Effect of calcination temperature and heating rate on the optical properties and reactivity of rice husk ash. J. Mater. Sci..

[30] Feng Q., Yamamichi H., Shoya M., Sugita S. (2004). Study on the pozzolanic properties of rice husk ash by hydrochloric acid pretreatment. Cem. Concr. Res..

